# Bulk-level maps of pioneer factor binding dynamics during the *Drosophila* maternal-to-zygotic transition

**DOI:** 10.1242/dev.204460

**Published:** 2025-07-03

**Authors:** Sadia Siddika Dima, Gregory T. Reeves

**Affiliations:** ^1^ Department of Chemical Engineering, Texas A&M University, College Station, TX 77843, USA; ^2^ Faculty in Genetics and Genomics, Texas A&M University, College Station, TX 77843, USA

**Keywords:** Pioneer factors, Zygotic genome activation, Zelda, GAGA factor, DNA binding, Raster image correlation spectroscopy

## Abstract

Gene regulation by transcription factors (TFs) binding cognate sequences is of paramount importance. For example, the TFs Zelda (Zld) and GAGA factor (GAF) are widely acknowledged for pioneering gene activation during zygotic genome activation (ZGA) in *Drosophila*. However, quantitative dose/response relationships between bulk TF concentration and DNA binding, an event tied to transcriptional activity, remain elusive. Here, we map these relationships during ZGA: a crucial step in metazoan development. To map the dose/response relationship between nuclear concentration and DNA binding, we performed raster image correlation spectroscopy, a method that can measure biophysical parameters of fluorescent molecules. We found that, although Zld concentration increases during nuclear cycles 10 to 14, its binding in the transcriptionally active regions decreases, consistent with its function as an activator for early genes. In contrast, GAF-DNA binding is nearly linear with its concentration, which sharply increases during the major wave, implicating its involvement in the major wave. This study provides key insights into the properties of the two factors and puts forward a quantitative approach that can be used for other TFs to study transcriptional regulation.

## Teaser

**Summary:** The early *Drosophila* pioneer factors Zelda and GAGA factor undergo complementary dynamics, with Zelda binding decreasing over time, and GAF first binding to high-affinity clusters, then to low-affinity sites.

## INTRODUCTION

In *Drosophila*, zygotic genome activation (ZGA) begins with the transcription of a handful of genes during its minor wave, followed by a major wave when thousands of genes are transcribed (Harrison et al., 2023; Pritchard and Schubiger, 1996; Schulz and Harrison, 2019; Vastenhouw et al., 2019). The transcription factor (TF) Zelda (Zld) has the ability to bind nucleosomal DNA (Garcia et al., 2019; McDaniel et al., 2019) and subsequently to facilitate the binding of other TFs (Satija and Bradley, 2012; Li et al., 2014; Foo et al., 2014; Sun et al., 2015; Schulz et al., 2015; Yáñez-Cuna et al., 2012; Xu et al., 2014), which are the two defining features of a special class of TFs known as pioneer factors (Iwafuchi-Doi and Zaret, 2014; Zaret and Carroll, 2011; Zaret and Mango, 2016). The maternally encoded TF GAGA factor (GAF) also possesses pioneer-like properties (Blythe and Wieschaus, 2015, 2016; Gaskill et al., 2021; Judd et al., 2021; Schulz et al., 2015; Sun et al., 2015).

The relationship between the concentration of TFs such as these pioneer factors and gene expression remains an open question (Ay and Arnosti, 2011; Kim and O'Shea, 2008). Fluorescent imaging of live and fixed tissues can give relative concentrations of TFs, which are often used to predict TF activity by assuming some nonlinear relationship between concentration and activity (Jaeger et al., 2004; Kanodia et al., 2012; Manu et al., 2009; O'Connell and Reeves, 2015; Papatsenko and Levine, 2005, 2011; Zinzen et al., 2006). However, validation of these relationships using dynamic quantitative data in live cells is currently lacking. Furthermore, the binding of the TFs to DNA – which is required for transcriptional regulation – is dependent not only on their nuclear concentration, but also on factors such as chromatin accessibility and saturation kinetics. On the other hand, recent studies have identified hubs of TF binding at DNA sites, which may provide more direct measures of TF activity (Dufourt et al., 2018; Mir et al., 2017, 2018; Yamada et al., 2019). Thus, knowing only total concentration is inadequate to predict TF binding, and direct measurements of TF hubs lack generalizability; an input/output map between the two, based on quantitative measurements of concentration and binding, is required.

To bridge this gap, we used raster image correlation spectroscopy (RICS) to quantify the concentration and binding of the pioneer-like factors Zld and GAF in live embryos over the course of nuclear cycles (ncs) 10-14 (Al Asafen et al., 2024; Brown et al., 2008; Digman and Gratton, 2009; Digman et al., 2005a,b; Schloop et al., 2025). These measurements allowed us to construct dose/response relationships suggesting that the binding of Zld to transcriptionally active sites is dependent on factors other than its concentrations alone, whereas the GAF concentration is the primary driver of its binding. Furthermore, we found that GAF must bind and saturate its sites in the inactive regions of the DNA before it can bind to active DNA sites, resulting in a delay in its role in ZGA. Similar approaches can be used to obtain a comprehensive quantitative picture of dynamics of other TFs for gene regulation studies.

## RESULTS AND DISCUSSION

**Fig. 1.**
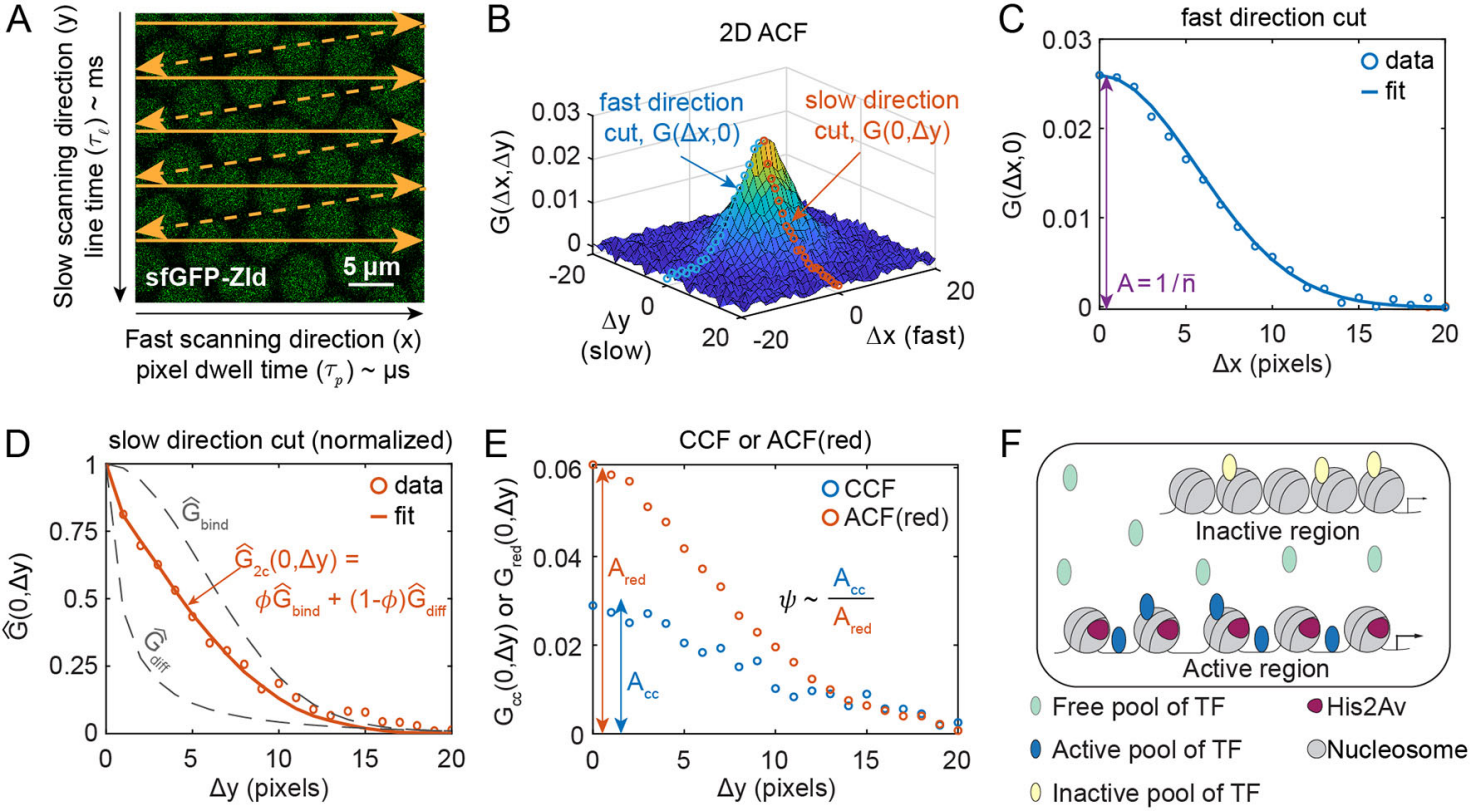
**Raster Image Correlation Spectroscopy (RICS).** (A) Laser scanning confocal microscopes build images by raster scan, with a fast scanning direction (*x* direction, solid arrows) and a slow scanning direction (*y* direction) due to line retracing (dashed arrows). Image shows a mid-nc14 embryo expressing sfGFP-Zld. (B) Two-dimensional autocorrelation function (ACF) from the embryo depicted in A. Cuts along the fast (blue circles) and slow (orange circles) directions are depicted. (C) Cut of ACF along the fast direction. Solid curve: fit of Gaussian-shaped PSF, used to estimate the ACF amplitude, *A*. (D) Plot of the slow direction data (circles) and the fit to the slow direction (solid curve), composed of a linear combination between two ACFs (gray dotted curves): an immobile ACF 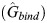 and a diffusible ACF 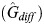. The linear combination weight is *φ*, the immobile fraction. (E) Plots of cuts of the cross correlation function (CCF; blue circles) and of the ACF in the red channel (orange circles). The ratio of the amplitudes (*A*_*cc*_/*A*_*red*_) is proportional to *ψ*, the fraction bound in active regions of the DNA. (F) Illustration of the different pools of TF: freely diffusible (light blue), bound to active regions (dark blue) and bound to inactive regions (yellow). His2Av (purple) is associated with the active regions of DNA.

Raster-scanned confocal images have a fast-scanning direction (pixel-to-pixel) and a slow-scanning direction (line retracing; Fig. 1A). RICS analysis uses intensity fluctuations of GFP-tagged molecules, correlated in time and space, to build an autocorrelation function (ACF) in two dimensions, corresponding to the fast (Δx) and slow (Δy) scanning directions, respectively (Fig. 1B; see Materials and Methods). The amplitude of the ACF, *A*, is inversely proportional to the GFP concentration (Fig. 1C), while the shape of the ACF, especially in the slow direction, is determined by the fraction of GFP that is freely diffusible versus that which is immobile (Fig. 1D). Furthermore, cross-correlations in the fluctuations between GFP and RFP (tagged to His2Av) allow us to calculate the fraction of GFP-tagged molecules that are bound to the same structure as the His2Av-RFP (likely to be DNA; Bacia and Schwille, 2007; Weidemann et al., 2002; Fig. 1E). Therefore, RICS allows us to quantify not only the absolute concentration of GFP-tagged molecules, but also the fraction that is freely diffusible, the immobile fraction, and the fraction correlated to His2Av (Fig. 1F).

### Zld levels bound to DNA decrease while nuclear concentration increases

**Fig. 2.**
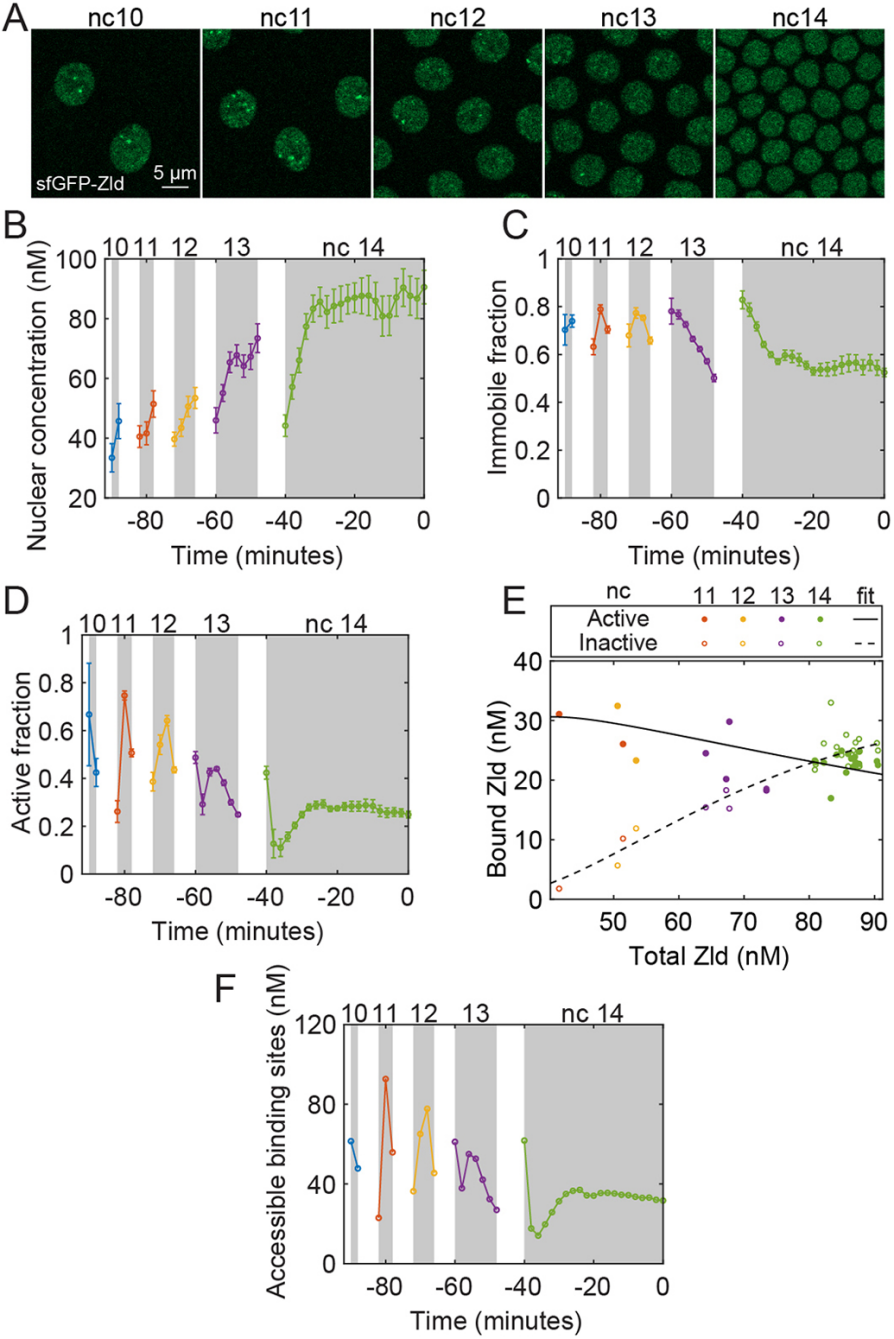
**Quantification of the biophysical parameters and dynamics of Zld.** (A) Representative images of a sfGFP-Zld embryo used for Raster Image Correlation Spectroscopy (RICS) analysis from nc 10 to nc 14, as indicated. (B-D) Dynamics of different pools of sfGFP-Zld from nc 10 until gastrulation, including total nuclear concentration (B), immobile fraction (C) and active fraction (D). Data are mean±s.e.m. (*n*=20 embryos). (E) Dose/response map between total nuclear concentration of Zld and the immobile (both active and inactive) concentration of Zld. The solid line represents the model fit for the active population and the dashed line represents the same for the inactive population. (F) Change in Zld sites accessible for binding over time. See also Fig. S1 and Movie 1.

We performed RICS analysis on the nuclei of blastoderm-stage embryos expressing sfGFP-Zld (Hamm et al., 2017) (Fig. 2A and Movie 1) to measure the dynamics of the nuclear concentration of Zld over time. We found the total nuclear concentration of Zld increased from one nuclear cycle to the next during nc 10 to 14 (Fig. 2B). Increase in Zld levels has been reported previously using immunoblotting (Harrison et al., 2010; Nien et al., 2011). Furthermore, the nuclear concentrations of Zld vary significantly within each nc, as more sfGFP-Zld enters nuclei after mitosis and fills it during the interphase. The longer duration of nc 14 allows Zld levels to reach a steady state, unlike the other, shorter, ncs. In contrast, we observed that the nuclear Zld concentration drastically decreases from the end of one interphase to the beginning of the next (during mitosis), in agreement with previous observations that, unlike most pioneer factors, Zld is not mitotically retained on the chromosomes (Dufourt et al., 2018).

RICS analysis also allowed us to determine *φ*, the fraction of Zld that is immobile (or nearly so) due to either binding to immobile structures (such as DNA) or to forming large aggregates with very low diffusivity (Eqn 4). The immobile fraction of Zld remains nearly the same from nc 10 to nc 13, after which it reaches a steady state at a lower value of ∼0.5 at nc 14 (Fig. 2C). Fifty percent of the Zld population was found to be immobile in single-molecule imaging (Mir et al., 2018).

In each nc, at the beginning of interphase, the immobile concentration of Zld, calculated as the product of total nuclear concentration and the immobile fraction, increases, perhaps because the chromatin decondenses and Zld binds the replicated DNA (Antonin and Neumann, 2016; Blythe and Wieschaus, 2016; McDaniel et al., 2019; Yuan et al., 2014), and it starts decreasing after reaching a maximum as chromatin starts condensing before mitosis (Fig. S1A). This is consistent with the rapid formation of dynamic hubs of Zld after mitosis (Mir et al., 2018), the hubs likely being a population included in our immobile concentration measurements. It should be noted that the presence of hubs results in Zld particles with higher brightness than the Zld monomer. As such, the presence of hubs, in principle, could alter our measurements of total Zld nuclear concentration and of the immobile fraction. We have quantified the average particle brightness and found it to be roughly constant over time, suggesting that hubs of Zld have little effect on our measurements (see Materials and Methods and Fig. S2).

We also used cross-correlation RICS (ccRICS) to determine *ψ*, the fraction of the pioneer factors that correlate with His2Av-RFP (Eqn 9 in the Materials and Methods). In *Drosophila*, the enrichment of the histone variant His2Av has been found to correlate with transcriptional potential (Leach et al., 2000; Mavrich et al., 2008), and therefore, the pool correlated with His2Av-RFP (hereafter referred to as the active pool) is likely responsible for pioneering gene activation. As the immobile fraction always measured as larger than the active fraction (Fig. 2C,D), we inferred that the immobile fraction must be composed of two pools: one that correlates with His2Av-RFP and one that does not. Hereafter, the pool that does not correlate with His2Av but is immobile, either due to binding to inactive chromatin regions or due to low diffusivity from the formation of large clusters, is referred to as the inactive pool. We found the active fraction decreases from nuclear cycle to nuclear cycle (Fig. 2D). Consequently, the active concentration of Zld, which is the product of the total nuclear concentration and the active fraction of Zld, also decreases, albeit weakly, due to the increasing total nuclear concentration (Fig. S1B). Our results agree with the slight reduction observed in the number of Zld peaks from nc 13 to nc 14 in ChIP-Seq (Harrison et al., 2011). The Zld ChIP-Seq peaks likely represent the binding in the active regions as tightly packed inactive regions are less likely to be identified (Nakato and Shirahige, 2016). Roughly 5 min into nc 14, the concentration of active Zld reaches a steady value around 20-25 nM (Fig. S1B).

Our measurements lend themselves to a general analysis in which the relationship between Zld total nuclear concentration and its binding could be constructed. According to standard thermodynamic equilibrium binding models (see Materials and Methods), an increase in the total Zld concentration should lead to an increase in the bound Zld concentration. However, our data suggest that the active concentration of Zld is a weakly decreasing function of total Zld (Fig. 2E) in which nc 10-12 has a high active concentration of Zld despite the low total Zld concentration, while in nc 14, there is a high total Zld concentration and a slightly lower active concentration. As such, our results suggest that Zld binding is not influenced by Zld concentration alone; additional factors, such as chromatin structure, must be taken into account. Using our data and ChIP-seq results reported by Harrison et al. (2011), the dissociation constant for the active population (*K*_*D*,*a*_) for Zld can be estimated to be ∼17 nM (see Materials and Methods). Fitting a Hill function to our data suggests that the Zld sites accessible for binding reduce over time (Fig. 2F), which might result from the chromatin becoming more densely packed during the later cycles (Harrison et al., 2011; Li et al., 2014; Lowenhaupt et al., 1983). On the other hand, the inactive concentration of Zld increases with time (Fig. S1C), suggesting the inactive fraction may result from binding that is saturating in nature. Fitting a Hill function with a Hill coefficient of 2, due to the sigmoidal nature of the data points, yielded the dissociation constant for the inactive population (*K*_*D*,*i*_) of ∼24 nM and a max concentration of 34 nM (Fig. 2E). These values of *K_D_* suggest that the binding of Zld to DNA or to immobile structures including aggregates is moderately strong. Overall, the dose/response relationship between total and bound Zld concentration is consistent with the previously observed hubs of Zld bound to DNA. Zld molecules within these hubs have short residence times on DNA (*τ*∼5 s; Mir et al., 2018). This is lower than our estimation (see Materials and Methods), which might result from the high local concentration of Zld within the hub (Mir et al., 2018) or from the local Zld diffusivity (Mirny et al., 2009).

Our data suggest that Zld may not retain its ability to pioneer chromatin accessibility throughout ZGA. Consistent with this, it was recently shown that, in larval type II neuroblasts, Zld binding is influenced by chromatin accessibility (Larson et al., 2021). Thus, our observation may imply that Zld primarily regulates transcription during the minor wave. Indeed, the removal of Zld activity affects early gene expression patterns, resulting in a delay in, but not complete loss of, the transcription of patterning genes (Liang et al., 2008; Nien et al., 2011). Therefore, our results suggest that, during the minor wave of ZGA (nc 10-13), a high concentration of bound Zld might be needed to facilitate the binding of patterning factors present at low levels. On the other hand, during the major wave at nc 14, either the high level of patterning factors, or the presence of other pioneer-like factors, may be sufficient to drive the patterning factor binding and target gene expression when the bound Zld levels have decreased. This raises the question of whether other factors are present during the major wave that may continue to facilitate the binding of other developmental TFs.

### GAF levels increase suddenly in nc 14

GAF, which is encoded by the *Trithorax-like* (*Trl*) gene, plays an essential role in ZGA, along with Zld (Farkas et al., 1994; Gaskill et al., 2021). GAF motifs along with Zld motifs are enriched in the highly occupied target (HOT) regions characterized by open chromatin and bound by many TFs (The modENCODE Consortium et al., 2011; Kvon et al., 2012; Slattery et al., 2014). GAF possesses the properties of a pioneer factor, such as the ability to bind nucleosomal DNA and to create regions of chromatin accessibility by functioning with chromatin remodelers to facilitate the binding of other TFs (Fuda et al., 2015; Gaskill et al., 2021; Judd et al., 2021; Moshe and Kaplan, 2017; Tang et al., 2022; Tsukiyama et al., 1994).

**Fig. 3.**
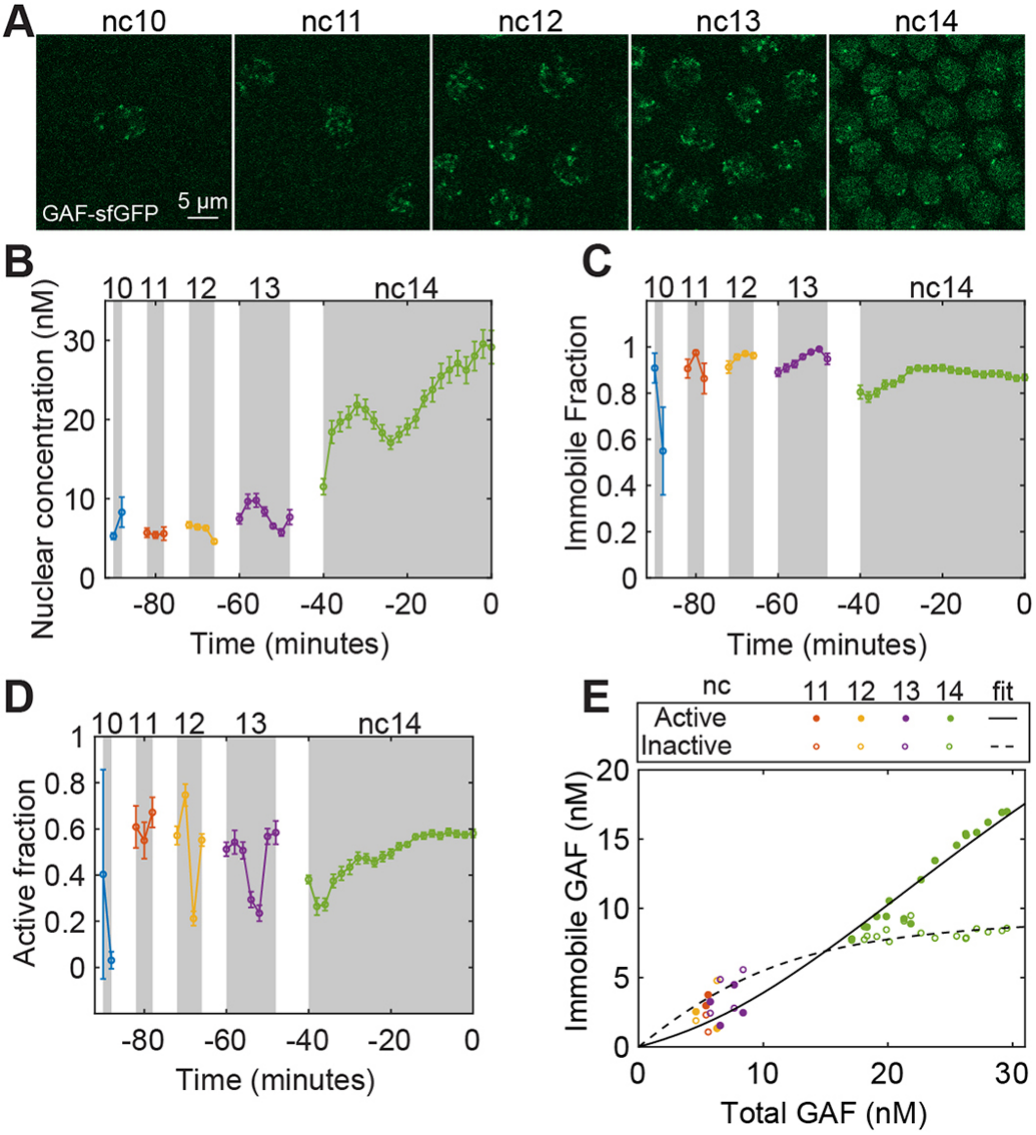
**Quantification of the biophysical parameters and dynamics of GAF.** (A) Representative images of a GAF-sfGFP(C) embryo used for Raster Image Correlation Spectroscopy (RICS) analysis from nc 10 to nc 14, as indicated. (B-D) Dynamics of the parameters from nc 10 until gastrulation, including total nuclear concentration (B), the immobile fraction (C) and the active fraction (D). Data are mean±s.e.m. (*n*=19 embryos). (E) Dose/response map between total nuclear concentration of GAF and the immobile (both active and inactive) concentration of GAF. The solid line represents the model fit for the active population and the dashed line represents the same for the inactive population. See also Fig. S3 and Movie 2.

To measure the dynamics of the nuclear concentration of GAF over time, we performed RICS analysis on the nuclei of blastoderm-stage embryos expressing GAF-sfGFP (Gaskill et al., 2021) (Fig. 3A and Movie 2). The results suggest that the total nuclear concentration of GAF remained nearly constant and very low (∼5-10 nM) from nc 10-13, then showed a sudden increase during nc 14 to ∼30 nM (Fig. 3B). We saw that the nuclear concentration at the beginning of an interphase was similar to that at the end of the previous interphase (Fig. 3B). This observation is consistent with previous work showing GAF is mitotically retained on the chromosomes (Gaskill et al., 2021), and is in contrast to Zld (Fig. 2B), which is not mitotically retained (Dufourt et al., 2018).

The immobile fraction of GAF remained nearly the same during nc 10-13, then decreased slightly during nc 14 (Fig. 3C). The sudden increase in GAF total nuclear concentration, together with only a slight decrease in its immobile fraction, resulted in an increase in the immobile concentration of GAF during nc 14 (Fig. S3A). Although the active fraction of GAF, the pool expected to be bound near the actively transcribed genes, remained approximately the same, on average, from nc 10-14, it varied significantly within each nc (Fig. 3D).

As with Zld, we sought to use our data to map the relationship between GAF nuclear concentration and binding. We noted that, unlike Zld, both active and inactive concentrations of GAF increase with an increase in the total concentration of GAF (Fig. 3E). Our results agree with the increase in GAF ChIP-seq peaks from nc 9 to 14 observed previously (Gaskill et al., 2021). Both pools appeared to be saturating functions of total GAF concentration, with the inactive pool saturating quickly at a low overall concentration (Fig. 3E and Fig. S3B,C). In contrast, the active pool required higher levels of total GAF to saturate and had a higher capacity. Fitting Hill functions to the data bore out these observations: the inactive pool had a *K*_*D*,*i*_ of 0.4 nM and a max concentration of 9 nM, while the active pool had a *K*_*D*,*a*_ of 5 nM and a max concentration of 35 nM. The *K_D_* values represent a strong affinity of GAF for the binding sites, roughly 4-fold and 60-fold greater than for Zld, consistent with previous reports of stable GAF/DNA binding (Bellec et al., 2022; Espinás et al., 1999; Tang et al., 2022). It appears that, as GAF concentration slowly increases, the majority is apportioned to the inactive pool due to its high affinity. However, because of the low capacity of the inactive pool, upon entering nc 14, the active pool is suddenly able to increase. If the active pool corresponds to transcriptionally active regions of the DNA (i.e. euchromatin), the relative affinities and capacities ensure that GAF acts as a pioneer factor solely during the later, major, wave of ZGA. Furthermore, it has been shown that GAF associates with the GA/CT-rich repeats in the heterochromatin regions throughout cell cycles (Gaskill et al., 2023; Raff et al., 1994), potentially driving transcriptional silencing and the euchromatin association activation during ZGA (Gaskill et al., 2023). As such, the inactive pool may represent GAF bound to heterochromatin. As with Zld, GAF hubs with higher brightness could affect our measurements. Quantification of the brightness suggested small corrections to our data, which did not affect our overall conclusions (see Materials and Methods and Fig. S4).

**Fig. 4.**
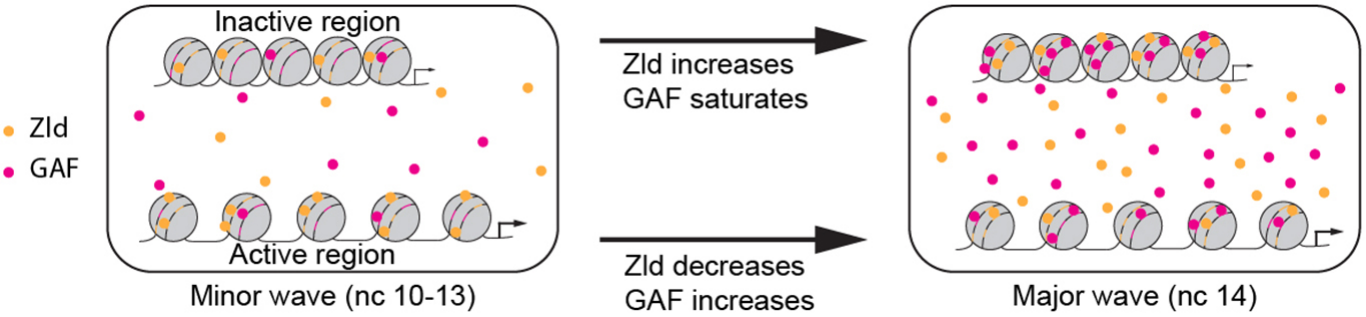
**Role of pioneer factors Zld and GAF during *Drosophila* ZGA.** During the minor wave of ZGA, a high concentration of Zld (yellow circles) bound to its sites (yellow lines) in the active regions allows it to act as the main activator of early genes. This active bound concentration reduces during the major wave, when the concentration of GAF (magenta circles) bound to the sites (magenta lines) in the active regions increases, after saturating the sites in the inactive regions. This allows GAF to act as the main activator of the genes that begin to be expressed during the major wave.

Our measurements of the biophysical parameters of the pioneer factors and their dynamics allowed the construction of input/output maps between total concentration of the factors and their DNA binding. While a subset of the pioneer factor bound regions has been found to stay inaccessible (Freund et al., 2024; Gibson et al., 2024), binding of pioneer factors to DNA is an indispensable step for establishing and maintaining chromatin accessibility. Following this, binding of a correct combination of TFs can lead to the activation of the accessible regions (Brennan et al., 2023). Using the input/output maps, we can infer that Zld acts as a global activator of early genes enabled by the high active concentration of Zld during the minor wave of ZGA; GAF comes into play during the major wave, as indicated by the high active concentration of GAF during this time (Fig. 4). This is supported by the fact that the regions that gain accessibility early during ZGA are enriched for Zld binding, whereas those that gain accessibility late are enriched for GAF binding (Blythe and Wieschaus, 2016). A similar quantitative approach, in which accurate measurements of biophysical parameters of TFs and their dynamics enable the construction of input/output map between TF concentration and transcriptional activity, is expected to aid other gene expression studies.

## MATERIALS AND METHODS

### *Drosophila* strains

The fly strain used for the Zld imaging is *sfGFP-zld; His2Av-RFP (II).* The *sfGFP-zld* mutant allele was generated using Cas9-mediated genome engineering by Hamm et al. (2017). The fly strain used for the GAF imaging is *His2Av-RFP (II); GAF-sfGFP(C) (III)*. The *GAF-sfGFP(C) (III)* mutant allele was generated using Cas9-mediated genome engineering by Gaskill et al. (2021).

### Sample preparation

Flies were raised on standard cornmeal-molasses-yeast medium at 25°C. Fly cages were prepared with desired fly strains and kept at room temperature for 2 days before imaging. Grape juice agar plates streaked with yeast paste were placed onto the bottoms of the cages for oviposition. For imaging, the flies were allowed to lay eggs for 1 h after which the plates were removed for embryo collection. The embryos were transferred from the plates to mesh baskets, dechorionated using bleach for 30 s and washed with deionized water to remove residual bleach (Carrell and Reeves, 2015).

### Live imaging

After dechorionation, the embryos were mounted in 1% low melting point agarose (IBI Scientific, IB70051) in deionized water on a glass bottom Petri dish (MatTek, P35G-1.5-20-C) and deionized water was poured into the Petri dish over the solidified low melting point agarose to cover the samples. The low melting point agarose rendered mechanical stability to the embryos while remaining transparent submerged in water (Huisken and Stainier, 2009; Kaufmann et al., 2012). All images were collected on a Zeiss LSM 900 confocal laser scanning microscope. For the image acquisitions, C-Apochromat 40×/1.2 water immersion Korr objective, 488 nm laser for sfGFP, 561 nm laser for RFP and GaAsP-PMT detector were used. The detector was operated at 650 V with 1× gain and 0% offset. Emission was detected in the range of 410-546 nm for sfGFP and 595-700 nm for RFP. All the images had 1024×1024 pixel resolution. The images were collected at 5× zoom, resulting in pixel size of 31.95 nm and a pixel dwell time of 2.06 µs. This corresponds to a frame time of 5.06 s and a line time of ∼5 ms (ratio of frame time and number of rows in the image). Image acquisition was started when the embryos were at nc 10 and continued until gastrulation, as indicated by the nuclear morphology.

### Raster image correlation spectroscopy image analysis

Raster image correlation spectroscopy (RICS) analysis, a derivative of fluorescence correlation spectroscopy, entails constructing autocorrelation functions (ACFs) from imaging data and fitting ACF models to these data-derived ACFs. We performed these analyses according to previous protocols (Al Asafen et al., 2024; Schloop et al., 2025). In brief, live imaging time courses were background subtracted and divided into groups of 7-12 frames. The frames within a group were averaged together to create an averaged frame for that group, which was used for two purposes. First, the nuclei in the averaged frame were segmented according to a watershed algorithm. This segmented nuclear mask was used for each frame in the group. Second, the immobile variation within each frame was then removed by subtracting by the averaged frame on a pixel-by-pixel basis, then the scalar average intensity value of the averaged frame was added back (Digman et al., 2005a,b). Two dimensional (2D) RICS autocorrelation functions of the nuclear fraction of each frame were built using a fast Fourier transform protocol in Matlab, and these ACFs were averaged together for all frames in a given group. The result was a time series of 2D ACFs, each of which corresponded to a given grouping of 7-12 frames. Background subtraction was performed on the fly by examining the histogram of intensities and fitting a Gaussian to the lowest intensity pixels in the image.

### Experimental determination of axial displacement

We mounted diffraction-limited TetraSpeck beads (0.1 µm, T7279, Invitrogen) in 1% low melting point agarose on a glass bottom Petri dish following the same protocol as the embryos. Similarly, the agarose was immersed in deionized water upon solidification. The microscope parameters, such as the pixel size and frame time, were identical as the RICS acquisitions. We acquired *z*-stacks with 0.05 µm distance between the slices. We acquired six *z*-stacks: three different locations in the agarose and two different batches of beads mounted in the agarose.

The beads were then detected in the average image of the *z*-stack. A 3D Gaussian was fitted to the intensity of the beads using fmincon. The centroid coordinates of each detected bead and the highest intensity *z*-plane for the corresponding bead were used as the initial guesses for the centers in *x*, *y* and *z* directions. This was carried out separately for the two channels (Fig. S5A,B). The difference between the centers (*x*, *y* and *z*) for the two channels, as determined from the fit, were calculated for each bead (Fig. S5C-D). The full width at half maximum (FWHM) in the *z*-direction was calculated separately for the two channels (Fig. S5E).

### Fitting RICS autocorrelation functions to estimate concentration and mobility

RICS autocorrelation functions (ACFs) generally contain two orthogonal pieces of information: the amplitude determines the concentration of the species and the shape determines the mobility. For a purely diffusing species, the theoretical, normalized ACF, 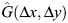, is the following:
(1)

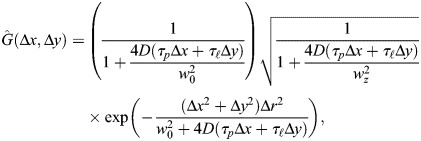
where *D* is the diffusivity. This equation has five microscope parameters: *w*_0_ and *w*_*z*_ are the radii of the point spread function (PSF) in the *xy* plane and the axial (*z*) direction, respectively; Δ*r* is the *xy* size of a pixel; and *τ*_*p*_ and *τ*_ℓ_ are the pixel dwell time (determined by the scan speed) and line time (determined by a combination of the scan speed and number of pixels in the width of the image), respectively. The two independent variables, Δ*x* and Δ*y*, are the pixel shifts in the fast and slow directions, respectively.

The non-normalized ACF includes the amplitude *A*, which is equal to the reciprocal of 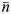 (the average number of molecules in the confocal volume):
(2)

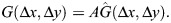
In practice, 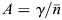, where 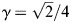 is a factor that accounts for the uneven illumination airy unit (Brown et al., 2008). Note that 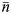 can be converted to 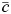, the average concentration in the confocal volume, *V*_*PSF*_, by 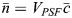, where 
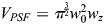
.

The 2D ACFs for sfGFP-Zld or GAF-sfGFP for each time point were then used to fit two different models. First, the fast direction cut of the 2D ACF, *G*_*s*_(Δ*x*, 0), was used to fit a Gaussian equation that approximates the PSF of the microscope. Because Δ*y*=0 along this cut, and 
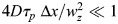
, Eqns 1 and 2 simplify to:
(3)

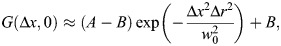
where, for robustness of fit, a small adjustable background constant, *B*, was added, and *w*_0_ was allowed to vary slightly. To avoid problems with background, *B* was constrained to have a magnitude less than 10^−3^; in practice, it never exceeded 1% of the value of the amplitude, *A*. This first fitting step resulted in accurate estimates of the ACF amplitude, *A*.

Second, holding *A* fixed, the entire 2D ACF was then used to fit a two component model, *G*_2*c*_(Δ*x*, Δ*y*), which is a linear combination of a freely diffusing fraction and an immobile fraction (Al Asafen et al., 2024):
(4)

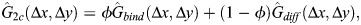
where 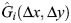 is given by Eqn 1 [with 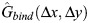 having *D*=0 and 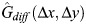 having *D* non-zero; see Fig. 1D], the linear combination weight, *φ*, is the immobile fraction and 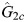 is the normalized two-component ACF, such that:
(5)

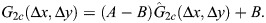
The parameter *B* in Eqn 5 may have a different value from the one found in Eqn 3. The 2D ACF for His2Av-RFP for each time point was used to fit only the 2D PSF to obtain a measure of the ACF amplitude.

### Fitting ccRICS cross-correlation functions to estimate correlated binding

Like the 2D ACF, the 2D cross-correlation function (CCF) between either sfGFP-Zld or GAF-sfGFP and His2Av-RFP was computed through a fast Fourier transform protocol in Matlab. This 2D CCF was then used to fit a 2D model of cross correlation (Al Asafen et al., 2024):
(6)

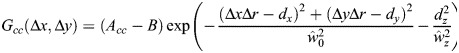
Because cross correlation uses two lasers, the two PSFs are different in size and their centers are generally not concurrent. In Eqn 6, the parameters 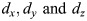 represent the displacements between the centers of the two PSFs in the *x, y* and *z* directions, respectively. The two displacements in the *xy* plane are adjustable parameters that can be estimated from the 2D CCF. The axial displacement was estimated by imaging fluorescent beads (see above), and our measurements resulted in the factor 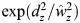 being roughly equal to 1.02, implying the factor could be safely ignored. As with the ACF, for robustness of fit, a small adjustable background constant, *B*, was added. Fitting Eqn 6 to the CCF data resulted in an estimate of the CCF amplitude, which is defined as:
(7)

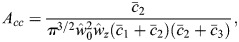
where the average PSF sizes are defined as 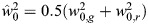 and 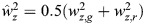, and the subscripts *g* and *r* denote the green and red channels, respectively, and where 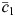 is the average concentration of the sfGFP-containing species that does not cross-correlate with the RFP-containing species (in the rest of the paper, this is the free pool plus the inactive pool); 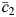 is the average concentration of the active pool and 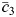 is the average concentration of the RFP-containing species that does not cross-correlate with the sfGFP-containing species, which include DNA-bound molecules, but could also include freely diffusing molecules.

To obtain *ψ*, which is the fraction of sfGFP-Zld or GAF-sfGFP correlated to His2Av-RFP, we calculate the ACF amplitude of the red channel, *G*_*red*_(Δ*x*, Δ*y*), then use it to fit Eqn 3, yielding the amplitude 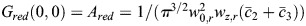. The ratio of *A*_*cc*_ to *A*_*red*_ then gives:
(8)

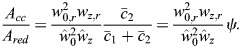
Therefore, we can calculate *ψ* as:
(9)

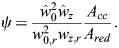


### Thermodynamic equilibrium model

When the binding of a TF to DNA is in equilibrium, the probability of the TF binding can be modeled as described by Bintu et al. (2005):
(10)

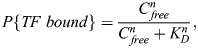
where *C_free_* is the nuclear concentration of the free TF, *K_D_* is the dissociation constant, *P*{*TF bound*}=*C*_*bound*_/*C*_*B*_, *C*_*bound*_ is the concentration of the bound TF and *C*_*B*_ is the concentration of binding sites. A Hill coefficient of *n*=1 was used in all the cases unless mentioned otherwise.

The equation was solved and adjustable parameters (dissociation constant and binding site concentrations) were determined using the least-squares fitting algorithm lsqcurvefit and the global optimization solver MultiStart in Matlab.

To express the concentration of active or inactive bound TF as a function of total nuclear concentration, we used the conservation relationship between different pools of the TF:
(11)

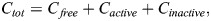
where *C_tot_* is the total nuclear concentration, *C_active_* is the concentration of the active pool and *C_inactive_* is the concentration of the inactive pool.

Using Eqn (10) this can be written as:
(12)

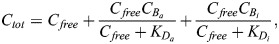
where 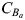 and 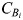 are the concentrations of binding sites for the active and inactive pools, respectively. 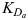 and 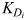 are the dissociation constants for the active and inactive pools, respectively.

In the case of Zld, 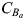 can be expressed as a function of *C_free_* (see Fig. S1D). Empirically, we modeled this relationship as a power law:
(13)

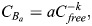
where *a*=420 and *k*=0.7 were the best-fit parameters (see Fig. S1D). The value of 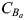 from Eqn 13 can be substituted into Eqn 12. Eqn 12 can then be used to determine the concentrations of free TF corresponding to a given total concentration to construct the dose/response maps in Figs 2E and 3E. For these calculations, nc 10 is ignored due to its short duration and data points near the start of each nc are excluded due to equilibrium not being established during these time points.

### Zld accessible sites

Pooled data from nc 8, 13 and 14 identified 12,135 Zld ChIP-Seq peaks (Harrison et al., 2011). We assumed that, during the early ncs, the chromatin is fairly open and all the ChIP-Seq identified sites are accessible for binding, such that the number of Zld sites accessible for binding during nc 11 is also assumed to be 12,135. Given that the nuclear volume during nc 11 is roughly 435 μm^3^ (Foe and Alberts, 1985), the concentration of Zld sites accessible for binding, averaged over the whole nucleus, is roughly, 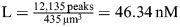. However, given that *Drosophila* is diploid, we set L=2×46.34=92.68 nM. Using these estimates, Eqn 10 and our data for nc 11, we arrive at *K_D_*_,*a*_=17 nM. Using this *K_D_* and our data in Eqn 10, the change in Zld sites accessible for binding over time was estimated.

### Residence time of Zld

Dissociation constants are typically defined as 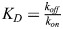; where *k_on_* and *k_off_* are the on and off rates for the TF-immobile structure interaction. For protein-ligand binding events in which the on-rate is limited by three dimensional diffusion, 10^−4^ nM^−1^ s^−1^<*k_on_*<10^−3^ nM^−1^ s^−1^ (Wittrup et al., 2019). Using this rule of thumb for the *k_on_* value and *K*_*D*_∼20 nM (valid for both the active and inactive pools of Zld), the residence time, 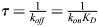, can be estimated to be *τ*∼8 min−50 s. However, if the diffusivity is higher, as would be the case of one-dimensional sliding along the DNA (Mirny et al., 2009), *k_on_* is closer to 10^−1^ nM^−1^ s^−1^, resulting in *τ*∼0.5 s. Owing to this variability using the rule of thumb, we used the Smoluchowski equation (Mirny et al., 2009) for the Zld-DNA interaction. The diffusivity of Zld obtained from our analysis (D) ∼2 µm^2^/s. According to the Smoluchowski equation (Mirny et al., 2009): *k*_*on*_=4*πDba*, where cross-section of the binding reaction (b)=0.34 nm and the fraction of the molecular surface of the protein that contains the reactive binding interface (a) ∼0.2-0.5. Therefore, *k_on_*∼0.001-0.003 nM^−1^ s^−1^, resulting in *τ*∼19−49 s, which agrees with the lower limit of the value calculated using the rule of thumb.

### Brightness analysis

The average molecular brightness, *Q*, of particles in the nucleus was calculated in the following manner. First, the average apparent brightness, *B*, of the sfGFP-containing particles was calculated as the ratio of the variance, *σ*^2^, of the intensity of pixels in the nuclei divided by their intensity, *I*:

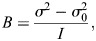
where 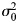 is the variance of a zero intensity image, which can also be found as the variance of the lowest intensity pixels in the image. Plots of *B* over nc 10-14 can be found in Figs S2A (Zld) and S4A (GAF).

Next, the *S*-factor, which relates apparent brightness and molecular brightness, was calculated as the slope of the best-fit line relating the microscope shot noise, 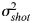 (which is found in the pixels in the nuclei) to the intensity of the nuclei (Dalal et al., 2008):

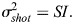


In practice, the shot noise is equal to the RICS ACF amplitude times the intensity squared, *AI*^2^, subtracted from the variance described above, 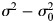. The shot noise and intensity at each time point in the time course was used to compute the best-fit line (see Fig. S2B for Zld and S4B for GAF). The equation used was 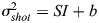, where *b* is a free parameter added for robustness of fit and was found generally to be close to zero. For Zld, *S*=363; for GAF, *S*=413.

Next, to convert the average apparent brightness into the average molecular brightness, the following relationship was used (Dalal et al., 2008):

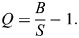
The plots of *Q* over nc 10-14 can be found in Figs S2C (Zld) and S4C (GAF).

Corrections for changes in brightness for GAF were performed in the following manner. First, a minimum value of *Q* was determined (*Q*_*min*_) and the brightness time course was normalized by *Q*_*min*_: *q*(*t*)=*Q*(*t*)/*Q*_*min*_, with values of *q*(*t*)<1 being set to 1. Next, new values of the RICS ACF amplitude, *A*_*new*_, and of the immobile fraction, *φ*_*new*_, were calculated from *q* and the old values (*A*_*old*_ and *φ*_*old*_):

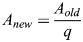


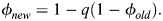


From these, corrected values of all concentrations, the free fraction and the uncorrelated fraction were calculated. These corrections were performed at two levels. In the first level, we assumed all variations within nc 14 were within the range of being roughly constant. In other words, only variations outside of this range were corrected for, meaning that *Q*_*min*_ was set as the maximum value observed during nc 14 (see Fig. S4C). This resulted in corrections made for the increase in brightness in the final point of nc 12 and the final four points of nc 13. While these corrections had a quantitative effect on our results, the corrected relationships between bound and free GAF are maintained (Fig. S4D,E).

## Supplementary Material



10.1242/develop.204460_sup1 Supplementary information

## References

[DEV204460C1] Al Asafen, H., Clark, N. M., Goyal, E., Jacobsen, T., Dima, S. S., Chen, H.-Y., Sozzani, R. and Reeves, G. T. (2024). Dorsal/NF-κB exhibits a dorsal-to-ventral mobility gradient in the Drosophila embryo. *eLife* 13, rp100462. 10.7554/eLife.100462.1

[DEV204460C2] Antonin, W. and Neumann, H. (2016). Chromosome condensation and decondensation during mitosis. *Curr. Opin. Cell Biol.* 40, 15-22. 10.1016/j.ceb.2016.01.013 26895139

[DEV204460C3] Ay, A. and Arnosti, D. N. (2011). Mathematical modeling of gene expression: a guide for the perplexed biologist. *Crit. Rev. Biochem. Mol. Biol.* 46, 137-151. 10.3109/10409238.2011.556597 21417596 PMC3086598

[DEV204460C4] Bacia, K. and Schwille, P. (2007). Practical guidelines for dual-color fluorescence cross-correlation spectroscopy. *Nat. Protoc.* 2, 2842-2856. 10.1038/nprot.2007.410 18007619

[DEV204460C5] Bellec, M., Dufourt, J., Hunt, G., Lenden-Hasse, H., Trullo, A., Zine El Aabidine, A., Lamarque, M., Gaskill, M. M., Faure-Gautron, H., Mannervik, M. et al. (2022). The control of transcriptional memory by stable mitotic bookmarking. *Nat. Commun.* 13, 1176. 10.1038/s41467-022-28855-y 35246556 PMC8897465

[DEV204460C6] Bintu, L., Buchler, N. E., Garcia, H. G., Gerland, U., Hwa, T., Kondev, J. and Phillips, R. (2005). Transcriptional regulation by the numbers: Models. *Curr. Opin Genet. Dev.* 15, 116-124. 10.1016/j.gde.2005.02.007 15797194 PMC3482385

[DEV204460C7] Blythe, S. A. and Wieschaus, E. F. (2015). Zygotic genome activation triggers the DNA replication checkpoint at the midblastula transition. *Cell* 160, 1169-1181. 10.1016/j.cell.2015.01.050 25748651 PMC4359640

[DEV204460C8] Blythe, S. A. and Wieschaus, E. F. (2016). Establishment and maintenance of heritable chromatin structure during early Drosophila embryogenesis. *eLife* 5, e20148. 10.7554/eLife.20148 27879204 PMC5156528

[DEV204460C9] Brennan, K. J., Weilert, M., Krueger, S., Pampari, A., Liu, H.-Y., Yang, A. W. H., Morrison, J. A., Hughes, T. R., Rushlow, C. A., Kundaje, A. et al. (2023). Chromatin accessibility in the Drosophila embryo is determined by transcription factor pioneering and enhancer activation. *Dev. Cell* 58, 1898-1916.e9. 10.1016/j.devcel.2023.07.007 37557175 PMC10592203

[DEV204460C10] Brown, C. M., Dalal, R. B., Hebert, B., Digman, M. A., Horwitz, A. R. and Gratton, E. (2008). Raster image correlation spectroscopy (RICS) for measuring fast protein dynamics and concentrations with a commercial laser scanning confocal microscope. *J. Microsc.* 229, 78-91. 10.1111/j.1365-2818.2007.01871.x 18173647 PMC3690660

[DEV204460C11] Carrell, S. N. and Reeves, G. T. (2015). Imaging the dorsal-ventral axis of live and fixed Drosophila melanogaster embryos. *Methods Mol. Biol.* 1189, 63-78. 10.1007/978-1-4939-1164-6_5 25245687

[DEV204460C12] Dalal, R. B., Digman, M. A., Horwitz, A. F., Vetri, V. and Gratton, E. (2008). Determination of particle number and brightness using a laser scanning confocal microscope operating in the analog mode. *Microsc. Res. Tech.* 71, 69-81. 10.1002/jemt.20526 17937391

[DEV204460C13] Digman, M. A. and Gratton, E. (2009). Analysis of diffusion and binding in cells using the RICS approach. *Microsc. Res. Tech.* 72, 323-332. 10.1002/jemt.20655 19067357 PMC4364519

[DEV204460C14] Digman, M. A., Brown, C. M., Sengupta, P., Wiseman, P. W., Horwitz, A. R. and Gratton, E. (2005a). Measuring fast dynamics in solutions and cells with a laser scanning microscope. *Biophys. J.* 89, 1317-1327. 10.1529/biophysj.105.062836 15908582 PMC1366616

[DEV204460C15] Digman, M. A., Sengupta, P., Wiseman, P. W., Brown, C. M., Horwitz, A. R. and Gratton, E. (2005b). Fluctuation correlation spectroscopy with a laser-scanning microscope: Exploiting the hidden time structure. *Biophys. J.* 88, L33-L36. 10.1529/biophysj.105.061788 15792971 PMC1305524

[DEV204460C16] Dufourt, J., Trullo, A., Hunter, J., Fernandez, C., Lazaro, J., Dejean, M., Morales, L., Nait-Amer, S., Schulz, K. N., Harrison, M. M. et al. (2018). Temporal control of gene expression by the pioneer factor Zelda through transient interactions in hubs. *Nat. Commun.* 9, 1-13. 10.1038/s41467-018-07613-z 30518940 PMC6281682

[DEV204460C17] Espinás, M. L., Jiménez-García, E., Vaquero, A., Canudas, S., Bernués, J. and Azorín, F. (1999). The N-terminal POZ domain of GAGA mediates the formation of oligomers that bind DNA with high affinity and specificity. *J. Biol. Chem.* 274, 16461-16469. 10.1074/jbc.274.23.16461 10347208

[DEV204460C18] Farkas, G., Gausz, J., Galloni, M., Reuter, G., Gyurkovics, H. and Karch, F. (1994). The Trithorax-like gene encodes the Drosophila GAGA factor. *Nature* 371, 806-808. 10.1038/371806a0 7935842

[DEV204460C19] Foe, V. E. and Alberts, B. M. (1985). Reversible chromosome condensation induced in Drosophila embryos by anoxia: visualization of interphase nuclear organization. *J. Cell Biol.* 100, 1623-1636. 10.1083/jcb.100.5.1623 3921555 PMC2113892

[DEV204460C20] Foo, S. M., Sun, Y., Lim, B., Ziukaite, R., O'Brien, K., Nien, C.-Y., Kirov, N., Shvartsman, S. Y. and Rushlow, C. A. (2014). Zelda potentiates morphogen activity by increasing chromatin accessibility. *Curr. Biol.* 24, 1341-1346. 10.1016/j.cub.2014.04.032 24909324 PMC4075064

[DEV204460C21] Freund, M. M., Harrison, M. M. and Torres-Zelada, E. F. (2024). Exploring the reciprocity between pioneer factors and development. *Development* 151, dev201921. 10.1242/dev.201921 38958075 PMC11266817

[DEV204460C22] Fuda, N. J., Guertin, M. J., Sharma, S., Danko, C. G., Martins, A. L., Siepel, A. and Lis, J. T. (2015). GAGA factor maintains nucleosome-free regions and has a role in RNA polymerase II recruitment to promoters. *PLoS Genet.* 11, e1005108. 10.1371/journal.pgen.1005108 25815464 PMC4376892

[DEV204460C23] Garcia, M. F., Moore, C. D., Schulz, K. N., Alberto, O., Donague, G., Harrison, M. M., Zhu, H. and Zaret, K. S. (2019). Structural features of transcription factors associating with nucleosome binding. *Mol. Cell* 75, 921-932.e6. 10.1016/j.molcel.2019.06.009 31303471 PMC6731145

[DEV204460C24] Gaskill, M. M., Gibson, T. J., Larson, E. D. and Harrison, M. M. (2021). GAF is essential for zygotic genome activation and chromatin accessibility in the early Drosophila embryo. *eLife* 10, e66668. 10.7554/eLife.66668 33720012 PMC8079149

[DEV204460C25] Gaskill, M. M., Soluri, I. V., Branks, A. E., Boka, A. P., Stadler, M. R., Vietor, K., Huang, H.-Y. S., Gibson, T. J., Mukherjee, A., Mir, M. et al. (2023). Localization of the Drosophila pioneer factor GAF to subnuclear foci is driven by DNA binding and required to silence satellite repeat expression. *Dev. Cell* 58, 1610-1624.e8. 10.1016/j.devcel.2023.06.010 37478844 PMC10528433

[DEV204460C26] Gibson, T. J., Larson, E. D. and Harrison, M. M. (2024). Protein-intrinsic properties and context-dependent effects regulate pioneer factor binding and function. *Nat. Struct. Mol. Biol.* 31, 548-558. 10.1038/s41594-024-01231-8 38365978 PMC11261375

[DEV204460C27] Hamm, D. C., Larson, E. D., Nevil, M., Marshall, K. E., Bondra, E. R. and Harrison, M. M. (2017). A conserved maternal-specific repressive domain in Zelda revealed by Cas9-mediated mutagenesis in Drosophila melanogaster. *PLoS Genet.* 13, e1007120. 10.1371/journal.pgen.1007120 29261646 PMC5752043

[DEV204460C28] Harrison, M. M., Botchan, M. R. and Cline, T. W. (2010). Grainyhead and Zelda compete for binding to the promoters of the earliest-expressed Drosophila genes. *Dev. Biol.* 345, 248-255. 10.1016/j.ydbio.2010.06.026 20599892 PMC2927720

[DEV204460C29] Harrison, M. M., Li, X.-Y., Kaplan, T., Botchan, M. R. and Eisen, M. B. (2011). Zelda binding in the early Drosophila melanogaster embryo marks regions subsequently activated at the maternal-to-zygotic transition. *PLoS Genet.* 7, e1002266. 10.1371/journal.pgen.1002266 22028662 PMC3197655

[DEV204460C30] Harrison, M. M., Marsh, A. J. and Rushlow, C. A. (2023). Setting the stage for development: the maternal-to-zygotic transition in Drosophila. *Genetics* 225, iyad142. 10.1093/genetics/iyad142 37616526 PMC10550319

[DEV204460C31] Huisken, J. and Stainier, D. Y. R. (2009). Selective plane illumination microscopy techniques in developmental biology. *Development* 136, 1963-1975. 10.1242/dev.022426 19465594 PMC2685720

[DEV204460C32] Iwafuchi-Doi, M. and Zaret, K. S. (2014). Pioneer transcription factors in cell reprogramming. *Genes Dev.* 28, 2679-2692. 10.1101/gad.253443.114 25512556 PMC4265672

[DEV204460C33] Jaeger, J., Surkova, S., Blagov, M., Janssens, H., Kosman, D., Kozlov, K. N., Manu, Myasnikova, E., Vanario-Alonso, C. E., Samsonova, M. et al. (2004). Dynamic control of positional information in the early Drosophila embryo. *Nature* 430, 368-371. 10.1038/nature02678 15254541

[DEV204460C34] Judd, J., Duarte, F. M. and Lis, J. T. (2021). Pioneer-like factor GAF cooperates with PBAP (SWI/SNF) and NURF (ISWI) to regulate transcription. *Genes Dev.* 35, 147-156. 10.1101/gad.341768.120 33303640 PMC7778264

[DEV204460C35] Kanodia, J. S., Liang, H.-L., Kim, Y., Lim, B., Zhan, M., Lu, H., Rushlow, C. A. and Shvartsman, S. Y. (2012). Pattern formation by graded and uniform signals in the early Drosophila embryo. *Biophys. J.* 102, 427-433. 10.1016/j.bpj.2011.12.042 22325264 PMC3274790

[DEV204460C36] Kaufmann, A., Mickoleit, M., Weber, M. and Huisken, J. (2012). Multilayer mounting enables long-term imaging of zebrafish development in a light sheet microscope. *Development* 139, 3242-3247. 10.1242/dev.082586 22872089

[DEV204460C37] Kim, H. D. and O'Shea, E. K. (2008). A quantitative model of transcription factor–activated gene expression. *Nat. Struct. Mol. Biol.* 15, 1192-1198. 10.1038/nsmb.1500 18849996 PMC2696132

[DEV204460C38] Kvon, E. Z., Stampfel, G., Yáñez-Cuna, J. O., Dickson, B. J. and Stark, A. (2012). HOT regions function as patterned developmental enhancers and have a distinct cis-regulatory signature. *Genes Dev.* 26, 908-913. 10.1101/gad.188052.112 22499593 PMC3347788

[DEV204460C39] Larson, E. D., Komori, H., Gibson, T. J., Ostgaard, C. M., Hamm, D. C., Schnell, J. M., Lee, C.-Y. and Harrison, M. M. (2021). Cell-type-specific chromatin occupancy by the pioneer factor Zelda drives key developmental transitions in Drosophila. *Nat. Commun.* 12, 7153. 10.1038/s41467-021-27506-y 34887421 PMC8660810

[DEV204460C40] Leach, T. J., Mazzeo, M., Chotkowski, H. L., Madigan, J. P., Wotring, M. G. and Glaser, R. L. (2000). Histone H2A.Z is widely but nonrandomly distributed in chromosomes of Drosophila melanogaster. *J. Biol. Chem.* 275, 23267-23272. 10.1074/jbc.M910206199 10801889

[DEV204460C41] Li, X.-Y., Harrison, M. M., Villalta, J. E., Kaplan, T. and Eisen, M. B. (2014). Establishment of regions of genomic activity during the Drosophila maternal to zygotic transition. *eLife* 3, e03737. 10.7554/eLife.03737 25313869 PMC4358338

[DEV204460C42] Liang, H.-L., Nien, C.-Y., Liu, H.-Y., Metzstein, M. M., Kirov, N. and Rushlow, C. (2008). The zinc-finger protein Zelda is a key activator of the early zygotic genome in Drosophila. *Nature* 456, 400-403. 10.1038/nature07388 18931655 PMC2597674

[DEV204460C43] Lowenhaupt, K., Cartwright, I. L., Keene, M. A., Zimmerman, J. L. and Elgin, S. C. R. (1983). Chromatin structure in pre- and postblastula embryos of Drosophila. *Dev. Biol.* 99, 194-201. 10.1016/0012-1606(83)90267-1 6413275

[DEV204460C44] Manu, Surkova, S., Spirov, A. V., Gursky, V. V., Janssens, H., Kim, A.-R., Radulescu, O., Vanario-Alonso, C. E., Sharp, D. H., Samsonova, M. et al. (2009). Canalization of gene expression in the Drosophila blastoderm by gap gene cross regulation. *PLoS Biol.* 7, e1000049. 10.1371/journal.pbio.1000049 19750121 PMC2653557

[DEV204460C45] Mavrich, T. N., Jiang, C., Ioshikhes, I. P., Li, X., Venters, B. J., Zanton, S. J., Tomsho, L. P., Qi, J., Glaser, R. L., Schuster, S. C. et al. (2008). Nucleosome organization in the Drosophila genome. *Nature* 453, 358-362. 10.1038/nature06929 18408708 PMC2735122

[DEV204460C46] McDaniel, S. L., Gibson, T. J., Schulz, K. N., Fernandez Garcia, M., Nevil, M., Jain, S. U., Lewis, P. W., Zaret, K. S. and Harrison, M. M. (2019). Continued activity of the pioneer factor Zelda is required to drive zygotic genome activation. *Mol. Cell* 74, 185-195.e4. 10.1016/j.molcel.2019.01.014 30797686 PMC6544384

[DEV204460C47] Mir, M., Reimer, A., Haines, J. E., Li, X.-Y., Stadler, M., Garcia, H., Eisen, M. B. and Darzacq, X. (2017). Dense Bicoid hubs accentuate binding along the morphogen gradient. *Genes Dev.* 31, 1784-1794. 10.1101/gad.305078.117 28982761 PMC5666676

[DEV204460C48] Mir, M., Stadler, M. R., Ortiz, S. A., Hannon, C. E., Harrison, M. M., Darzacq, X. and Eisen, M. B. (2018). Dynamic multifactor hubs interact transiently with sites of active transcription in Drosophila embryos. *eLife* 7, e40497. 10.7554/eLife.40497 30589412 PMC6307861

[DEV204460C49] Mirny, L., Slutsky, M., Wunderlich, Z., Tafvizi, A., Leith, J. and Kosmrlj, A. (2009). How a protein searches for its site on DNA: the mechanism of facilitated diffusion. *J. Phys. A Math. Theor.* 42, 434013. 10.1088/1751-8113/42/43/434013

[DEV204460C50] Moshe, A. and Kaplan, T. (2017). Genome-wide search for Zelda-like chromatin signatures identifies GAF as a pioneer factor in early fly development. *Epigenet. Chromatin* 10, 33. 10.1186/s13072-017-0141-5 PMC5496641 28676122

[DEV204460C51] Nakato, R. and Shirahige, K. (2016). Recent advances in ChIP-seq analysis: from quality management to whole-genome annotation. *Brief. Bioinform.* 18, 279-290. 10.1093/bib/bbw023 PMC5444249 26979602

[DEV204460C52] Nien, C.-Y., Liang, H.-L., Butcher, S., Sun, Y., Fu, S., Gocha, T., Kirov, N., Manak, J. R. and Rushlow, C. (2011). Temporal coordination of gene networks by Zelda in the early Drosophila embryo. *PLoS Genet.* 7, e1002339. 10.1371/journal.pgen.1002339 22028675 PMC3197689

[DEV204460C53] O'Connell, M. D. and Reeves, G. T. (2015). The presence of nuclear cactus in the early Drosophila embryo may extend the dynamic range of the dorsal gradient. *PLoS Comput. Biol.* 11, e1004159. 10.1371/journal.pcbi.1004159 25879657 PMC4400154

[DEV204460C54] Papatsenko, D. and Levine, M. (2005). Gene regulatory networks special feature: quantitative analysis of binding motifs mediating diverse spatial readouts of the Dorsal gradient in the Drosophila embryo. *Proc. Natl. Acad. Sci. USA* 102, 4966-4971. 10.1073/pnas.0409414102 15795372 PMC555988

[DEV204460C55] Papatsenko, D. and Levine, M. (2011). The Drosophila gap gene network is composed of two parallel toggle switches. *PLoS ONE* 6, e21145. 10.1371/journal.pone.0021145 21747931 PMC3128594

[DEV204460C56] Pritchard, D. K. and Schubiger, G. (1996). Activation of transcription in Drosophila embryos is a gradual process mediated by the nucleocytoplasmic ratio. *Genes Dev.* 10, 1131-1142. 10.1101/gad.10.9.1131 8654928

[DEV204460C57] Raff, J. W., Kellum, R. and Alberts, B. (1994). The Drosophila GAGA transcription factor is associated with specific regions of heterochromatin throughout the cell cycle. *EMBO J.* 13, 5977-5983. 10.1002/j.1460-2075.1994.tb06943.x 7813435 PMC395573

[DEV204460C58] Satija, R. and Bradley, R. K. (2012). The TAGteam motif facilitates binding of 21 sequence-specific transcription factors in the Drosophila embryo. *Genome Res.* 22, 656-665. 10.1101/gr.130682.111 22247430 PMC3317148

[DEV204460C59] Schloop, A. E., Hiremath, S. V., Shaikh, R., Dima, S. S., Lizardo, L., Bhakta, A., Williams, C. M. and Reeves, G. T. (2025). Spatiotemporal dynamics of NF-κB/Dorsal inhibitor IκBα/Cactus in Drosophila blastoderm embryos. *iScience* 112854. 10.1016/j.isci.2025.112854 40662193 PMC12256344

[DEV204460C60] Schulz, K. N. and Harrison, M. M. (2019). Mechanisms regulating zygotic genome activation. *Nat. Rev. Genet.* 20, 221-234. 10.1038/s41576-018-0087-x 30573849 PMC6558659

[DEV204460C61] Schulz, K. N., Bondra, E. R., Moshe, A., Villalta, J. E., Lieb, J. D., Kaplan, T., McKay, D. J. and Harrison, M. M. (2015). Zelda is differentially required for chromatin accessibility, transcription factor binding, and gene expression in the early Drosophila embryo. *Genome Res.* 25, 1715-1726. 10.1101/gr.192682.115 26335634 PMC4617967

[DEV204460C62] Slattery, M., Ma, L., Spokony, R. F., Arthur, R. K., Kheradpour, P., Kundaje, A., Nègre, N., Crofts, A., Ptashkin, R., Zieba, J. et al. (2014). Diverse patterns of genomic targeting by transcriptional regulators in Drosophila melanogaster. *Genome Res.* 24, 1224-1235. 10.1101/gr.168807.113 24985916 PMC4079976

[DEV204460C63] Sun, Y., Nien, C.-Y., Chen, K., Liu, H.-Y., Johnston, J., Zeitlinger, J. and Rushlow, C. (2015). Zelda overcomes the high intrinsic nucleosome barrier at enhancers during Drosophila zygotic genome activation. *Genome Res.* 25, 1703-1714. 10.1101/gr.192542.115 26335633 PMC4617966

[DEV204460C64] Tang, X., Li, T., Liu, S., Wisniewski, J., Zheng, Q., Rong, Y., Lavis, L. D. and Wu, C. (2022). Kinetic principles underlying pioneer function of GAGA transcription factor in live cells. *Nat. Struct. Mol. Biol.* 29, 665-676. 10.1038/s41594-022-00800-z 35835866 PMC10177624

[DEV204460C65] The modENCODE Consortium, Roy, S., Ernst, J., Kharchenko, P. V., Kheradpour, P., Negre, N., Eaton, M. L., Landolin, J. M., Bristow, C. A., Ma, L. et al. (2011). Identification of functional elements and regulatory circuits by Drosophila modENCODE. *Science* 330, 1787-1797. 10.1126/science.1198374 PMC3192495 21177974

[DEV204460C66] Tsukiyama, T., Becker, P. B. and Wu, C. (1994). ATP-dependent nucleosome disruption at a heat-shock promoter mediated by binding of GAGA transcription factor. *Nature* 367, 525-532. 10.1038/367525a0 8107823

[DEV204460C67] Vastenhouw, N. L., Cao, W. X. and Lipshitz, H. D. (2019). The maternal-to-zygotic transition revisited. *Development* 146, dev161471. 10.1242/dev.161471 31189646

[DEV204460C68] Weidemann, T., Wachsmuth, M., Tewes, M., Rippe, K. and Langowski, J. (2002). Analysis of ligand binding by two-colour fluorescence cross-correlation spectroscopy. *Single Mol.* 3, 49-61. 10.1002/1438-5171(200204)3:1<49::AID-SIMO49>3.0.CO;2-T

[DEV204460C69] Wittrup, K. D., Tidor, B., Hackel, B. J. and Sarkar, C. A. (2019). *Quantitative Fundamentals of Molecular and Cellular Bioengineering*. Cambridge, MA: The MIT Press.

[DEV204460C70] Xu, Z., Chen, H., Ling, J., Yu, D., Struffi, P. and Small, S. (2014). Impacts of the ubiquitous factor Zelda on Bicoid-dependent DNA binding and transcription in Drosophila. *Genes Dev.* 28, 608-621. 10.1101/gad.234534.113 24637116 PMC3967049

[DEV204460C71] Yamada, S., Whitney, P. H., Huang, S.-K., Eck, E. C., Garcia, H. G. and Rushlow, C. A. (2019). The Drosophila pioneer factor Zelda modulates the nuclear microenvironment of a dorsal target enhancer to potentiate transcriptional output. *Curr. Biol.* 29, 1387-1393.e5. 10.1016/j.cub.2019.03.019 30982648 PMC6702943

[DEV204460C72] Yáñez-Cuna, J. O., Dinh, H. Q., Kvon, E. Z., Shlyueva, D. and Stark, A. (2012). Uncovering cis-regulatory sequence requirements for context-specific transcription factor binding. *Genome Res.* 22, 2018-2030. 10.1101/gr.132811.111 22534400 PMC3460196

[DEV204460C73] Yuan, K., Shermoen, A. W. and O'Farrell, P. H. (2014). Illuminating DNA replication during Drosophila development using TALE-lights. *Curr. Biol.* 24, R144-R145. 10.1016/j.cub.2014.01.023 24556431 PMC3977024

[DEV204460C74] Zaret, K. S. and Carroll, J. S. (2011). Pioneer transcription factors: establishing competence for gene expression. *Genes Dev.* 25, 2227-2241. 10.1101/gad.176826.111 22056668 PMC3219227

[DEV204460C75] Zaret, K. S. and Mango, S. E. (2016). Pioneer transcription factors, chromatin dynamics, and cell fate control. *Curr. Opin. Genet. Dev.* 37, 76-81. 10.1016/j.gde.2015.12.003 26826681 PMC4914445

[DEV204460C76] Zinzen, R. P., Senger, K., Levine, M. and Papatsenko, D. (2006). Computational models for neurogenic gene expression in the Drosophila embryo. *Curr. Biol.* 16, 1358-1365. 10.1016/j.cub.2006.05.044 16750631

